# Nuclear factor erythroid 2-related factor 2 gene expression in patients with heat stroke and its association with oxidative stress and inflammation: a prospective study

**DOI:** 10.3389/fimmu.2026.1719289

**Published:** 2026-04-16

**Authors:** Guodong Lin, Haiyang Guo, Bingling Yin, Chongxiao Xu, Ting Chen, Yueli Zhao, Qiang Wen, Yu Shao, Zhiguo Pan

**Affiliations:** 1 Department of Graduate School, Guangzhou University of Chinese Medicine, Guangzhou, Guangdong, China; 2 Department of Critical Care Medicine, General Hospital of Southern Theatre Command of Chinese People's Liberation Army (PLA), Guangzhou, Guangdong, China; 3 The First Clinical Medical College, Southern Medical University, Guangzhou, Guangdong, China; 4 Department of Graduate School, South China University of Technology, Guangzhou, Guangdong, China; 5 Department of Ultrasound Medicine,The First Affiliated Hospital of University of Science and Technology of China (USTC), Hefei, China; 6 Department of Emergency Medicine, Weifang People’s Hospital, Weifang, Shandong, China; 7 Department of General Medicine, General Hospital of Southern Theatre Command of Chinese People's Liberation Army (PLA), Guangzhou, Guangdong, China; 8 Department of Emergency Medicine, General Hospital of Southern Theatre Command of Chinese People's Liberation Army (PLA), Guangzhou, Guangdong, China

**Keywords:** heatstroke, inflammation, multi-omics analysis, NRF2, oxidative stress

## Abstract

**Introduction:**

Heat strokes represent a critical health issue with high mortality when severe, yet treatments are limited and do not target core pathogenic mechanisms. Oxidative stress is instrumental in heat stroke progression and is closely related to inflammatory activation and endothelial damage. Nuclear factor erythroid 2-related factor 2 (NRF2) is a key antioxidant signaling molecule in sepsis, ischemia-hypoxic encephalopathy, and trauma, although its potential role(s) in heat stroke is unknown.

**Methods:**

From January 2023 to June 2025, we enrolled patients with heat stroke and healthy volunteers at the General Hospital of the Southern Theater Command of the Chinese People’s Liberation Army. Participants were divided into mild/severe heat stroke and control groups. Data collection included demographic characteristics, vital signs, disease-severity scores (Acute Physiology and Chronic Health Evaluation II [APACHE II] and Sequential Organ Failure Assessment [SOFA]), laboratory parameters (creatinine, total bilirubin, white blood cell count, procalcitonin, C-reactive protein, D-dimer, creatine kinase, prothrombin time, and platelet counts), and outcome measures (length of hospitalization and mortality).

**Results:**

We enrolled 49 patients with heat stroke (34 mild cases and 15 severe cases). Mortality did not differ between groups, but hospital stays were significantly longer in the severe group (11 days [range, 9–56]). Disease severity was greater in severe cases (APACHE II: 20 [13–24], SOFA: 10 [6–14]; p < 0.0001). Inflammation and organ-dysfunction markers were significantly higher in severe heat stroke group. Transcriptomic analysis showed that NRF2 and its downstream gene NAD(P)H quinone oxidoreductase 1 (*NQO1*) were expressed at significantly lower levels in patients with severe heat stroke compared than in healthy controls and mild cases. Blood malondialdehyde, advanced protein oxidation product, protein carbonyl, 8-hydroxydeoxyguanosine, and interleukin-6 levels in patients with severe heat stroke were significantly elevated. Correlation analysis showed that NRF2 expression correlated significantly with disease severity.

**Discussion:**

Patients with severe heat stroke demonstrated more extensive organ damage, oxidative stress, and inflammatory injury than those with mild heat stroke and healthy controls. Early after severe heat stroke, NRF2 and its downstream antioxidants HO-1 and NQO1 were significantly downregulated, suggesting that impaired NRF2-mediated antioxidant defense promotes disease pathogenesis.

## 1 Introduction

The global warming trend remains unchanged; thus, heat stroke is an important condition requiring attention in the future (1). Currently, few effective and specific treatments exist for severe heat stroke. Available approaches include broad-spectrum anti-inflammatory agents such as ulinastatin, extracorporeal blood-purification therapies, treatment for disseminated intravascular coagulation, blood transfusion, anti-rhabdomyolysis treatment, liver protection, and other organ-supportive measures. However, none of these strategies target the core pathogenic mechanisms underlying heat stroke (2). Several key findings, along with our own clinical experience, indicate that beyond aseptic inflammation and endothelial injury, oxidative stress is a critical driver of tissue damage during heat stroke. Moreover, oxidative stress interacts strongly with inflammatory processes and endothelial dysfunction (3–5). Therefore, in addition to anti-inflammatory interventions, developing novel and effective targets for antioxidant therapy holds significant clinical value. Nuclear factor erythroid 2-related factor 2 (NRF2) is a major antioxidant signaling molecule that are essential in various conditions, including sepsis, ischemic–hypoxic encephalopathy, and trauma (6). Nevertheless, its role in heat stroke remains unclear.

## 2 Materials and methods

### 2.1 Patient enrollment and classification

We admitted 49 patients with heat stroke to the intensive care unit and Department of Emergency Medicine of the General Hospital of the Southern Theater Command of the Chinese People’s Liberation Army between January 2023 and June 2025. The inclusion criteria included a confirmed diagnosis of heat stroke, an age of ≥ 18 years, and the availability of complete clinical data. Patients were excluded if they could not cooperate with treatment or if they had a hematological disease, an autoimmune disease, or abnormal coagulation function. In addition, we recruited 20 healthy volunteers as controls. The Ethics Committee of the General Hospital of the Southern Theater Command of the Chinese People’s Liberation Army approved the study protocol.

For each patient with heat stroke, data collection included demographic characteristics such as the admission time, age, and sex; vital signs including the heart rate, respiratory rate, body temperature, level of consciousness, use of vasoactive drugs, and need for endotracheal intubation; and disease severity as measured by the Acute Physiology and Chronic Health Evaluation II (APACHE II) score and the Sequential Organ Failure Assessment (SOFA) score. Laboratory parameters included creatinine, total bilirubin, white blood cell count, procalcitonin, C-reactive protein, D-dimer, creatine kinase, prothrombin time, and platelet count. Outcome indicators consisted of the length of hospital stay and mortality.

### 2.2 Diagnostic criteria for heat stroke

The diagnosis of heat stroke was established according to the Expert Consensus on Emergency Diagnosis and Treatment of Heat Stroke (2021 edition).

### 2.3 Treatment of heat stroke

Patient management was carried out in accordance with the Expert Consensus on Emergency Diagnosis and Treatment of Heat Stroke (2021 edition). After admission, patients received symptomatic and supportive care, including continuous monitoring of vital signs, active cooling, early fluid resuscitation, maintenance of electrolyte balance, organ protection, and nutritional support.

### 2.4 Grouping

Based on the diagnostic criteria employed, the enrolled patients were classified into a mild heat stroke group (n = 34) and severe heat stroke group (n = 15). In addition, 20 healthy volunteers were recruited to serve as a healthy control group (n = 20).

### 2.5 Experimental methods

#### 2.5.1 Isolation and extraction of peripheral blood mononuclear cells

PBMCs were isolated using density-gradient centrifugation with a lymphocyte-isolation solution. As blood cells have different densities, centrifugation separates them into distinct layers according to their specific gravity. To ensure optimal separation, both Ficoll solution and phosphate-buffered saline were equilibrated to 18-22°Cbefore use. Peripheral blood was first diluted with phosphate-buffered saline at a 1:1 ratio, ensuring thorough mixing. Ficoll solution was mixed gently via inversion, the lid was opened, and a sterile syringe was used to introduce a small amount of air to balance the pressure. An appropriate volume of Ficoll solution was then added to the bottom of a new 15 mL centrifuge tube because smaller tubes are more effective than 50 mL tubes for this procedure. The diluted peripheral blood was carefully layered on top of the Ficoll solution at a 1:1 ratio. Centrifugation was performed at 400 × *g* for 40 min at room temperature, with deceleration set to 0 and acceleration set to 2. Following centrifugation, four distinct layers formed from bottom to top, namely erythrocyte, Ficoll, PBMC, and plasma/platelet layers. The PBMC layer was carefully aspirated using a pipette. The collected PBMCs were washed by adding five times their volume of phosphate-buffered saline, followed by centrifugation at 100 × *g* for 10 min. The supernatant was discarded, and the cells were washed a second time in the same manner. The final pellet constituted purified PBMCs. A portion of these cells was sent for RNA sequencing, whereas the remaining PBMCs were used for reverse transcription-quantitative PCR (RT-qPCR) analysis.

#### 2.5.2 Enzyme-linked immunosorbent assay analysis

Serum interleukin (IL)-6 and IL-1β concentrations via ELISA analysis. Wash buffer, standard working solution, biotinylated antibody-working solution, and horseradish peroxidase-conjugate working solution were prepared in advance. Samples or standards were added to individual 96 wells and incubated at 37°C for 90 min. Each supernatant was then discarded without washing, and the biotinylated antibody working solution was added. After incubation at 37°C for 1 h, the wells were emptied and washed once. Horseradish peroxidase-labeled avidin was then added, followed by incubation at 37 °C for 30 min. The wells were washed five times, then tetramethylbenzidine substrate solution was added. After incubation at 37°C for 15 min, the stop solution was added, and optical density (OD) values at 450 nm were measured using a microplate reader. The IL-6 and IL-1β concentrations were determined using standard curves.

#### 2.5.3 RT-qPCR analysis

Total RNA was extracted from PBMCs using an RNA extraction kit, and RNA concentrations were measured. Complementary DNA was synthesized from the RNA using a reverse transcription kit. Each 20 μL reaction consisted of 10 μL of 2× qPCR reagent, 2 μL of complementary DNA (cDNA) template, 0.4 μL of forward primer (10 μmol/L), 0.4 μL of reverse primer (10 μmol/L), and 7.2 μL of diethyl pyrocarbonate-treated water. The real-time qPCR conditions were as follows: initial denaturation at 95 °C for 30 s, followed by 40 cycles of denaturation at 95 °C for 10 s, annealing at 60 °C for 10 s, and extension at 72 °C for 30 s. Glyceraldehyde-3-phosphate dehydrogenase messenger RNA (mRNA) was detected as an internal reference. The mRNA-expression levels of *NRF2* and genes encoding its downstream pathway signaling molecules heme oxygenase-1 (*HO-1*) and NAD(P)H quinone oxidoreductase 1 (*NQO1*) were calculated using the 2^−^ΔΔCt method.

#### 2.5.4 Transcriptomics analysis

For RNA-extraction and quality control (QC) analysis, we extracted total RNA from PBMCs using the TRNzol Universal Reagent (catalog number DP424). The RNA integrity and concentration were assessed using an Agilent 2100 bioanalyzer (Agilent Technologies, USA).

For library construction and sequencing, mRNA was enriched from total RNA using Oligo dT magnetic beads. Libraries were prepared using the Fast RNA-seq Lib Prep Kit V2 (catalog number RK20306, ABclonal, China). For strand-specific libraries, dUTP was incorporated instead of dTTP when synthesizing second-strand cDNA. The standard library-construction process included mRNA fragmentation, first- and second-strand cDNA synthesis, end repair, addition of A-tails, adapter ligation, fragment selection, and PCR amplification. After the constructed library passed the Qubit quantification and bioanalyzer test, an Illumina NovaSeq X Plus sequencer (Illumina, USA) was used for paired-end sequencing based on the sequencing-by-synthesis principle.

For bioinformatics analysis, raw sequencing data were quality-checked using FastP software, and the adaptor sequences and low-quality reads were removed to obtain clean reads. Clean reads were matched to the reference genome using HISAT2 (v2.0.5). Gene-expression levels were quantified using featureCounts (v1.5.0-p3) and normalized using fragments per kilobase of transcript per million mapped reads values.

For differential expression and functional analysis, DESeq2 (v1.20.0) was used for samples with biological duplicates, whereas edgeR (v3.22.5) was used with samples without replicates for normalization and difference analysis. Genes with a corrected p-value (expressed as the false-discovery rate [FDR]) of ≤ 0.05 and |log_2_ (fold-change [FC])| ≥ 1 were considered significantly differentially expressed. Gene Ontology (GO) and Kyoto Encyclopedia of Genes and Genomes (KEGG) pathway enrichment analysis were performed using clusterProfiler (v3.8.1) for differentially expressed genes. In parallel, gene set enrichment analysis (GSEA) was used to evaluate the enrichment of the predefined gene set. Protein–protein interaction networks of differentially expressed proteins were constructed and analyzed using the Search Tool for the Retrieval of Interacting Genes/Proteins (STRING) database.

#### 2.5.5 Proteomics analysis

Blood samples from patients with heat stroke were collected, and plasma, serum, and PBMCs were first treated with a high-abundance-protein removal column to enrich for low-abundance proteins. Each serum sample was incubated with the removal-column resin for 10 min at room temperature, then the penetrating solution was collected after centrifugation to obtain the protein extract.

The protein concentration of each sample was determined using the Bradford method and verified via 12% sodium dodecyl sulfate-polyacrylamide gel electrophoresis. The quantified protein was taken and dissolved in lysate (8 M urea, 100 mM triethylammonium bicarbonate, pH 8.5), then digested with trypsin at 37°C after reduction and alkylation (4 h and overnight). The enzymatically hydrolyzed peptides were washed on C18 StageTip desalting columns, eluted in 0.1% formic acid and 70% acetonitrile, and lyophilized for subsequent use.

Peptide reconstitution was performed using a nanoliter ultra-performance liquid chromatography system (Vanquish Neo, Thermo) with C18 pre-columns (catalog number 174500, 5 × mm, 300 μm, 5 μm) and C18 analytical columns (catalog number ES906, PepMap Neo UHPLC 150 μm × 15 cm, 2 μm) at a column temperature of 50°C, mobile phase A comprising 0.1% formic acid in water, and mobile phase B comprising 0.1% formic acid in 80% acetonitrile. Mass spectrometry analysis was performed using an Orbitrap Astral mass spectrometer (Thermo) in data-independent acquisition (DIA) mode via electrospray ionization (spray voltage: of 2.0 kV), with an ion-transport tube temperature of 290°C. The primary stage involved a full-scan resolution of 240,000 (mass: charge [m/z] ratio of 380–980), and the second stage involved 300 DIA windows with a resolution of 80,000.

The original data were searched and analyzed using DIA-NN software, with the following parameters: precursor mass tolerance of 10 parts per million (ppm), a fragment mass tolerance of 0.02 Da, fixed modification cysteine alkylation, and variable modification, such as N-terminal acetylation and methionine oxidation. The results were filtered at a 1% FDR. The screening criteria for identifying differentially expressed proteins were an FC in expression of > 1.2 or < 0.83. GO, InterPro, KEGG annotation, enrichment analysis and STRING protein-interaction network predictions were performed.

#### 2.5.6 Non-targeted metabolomics analysis

Serum samples were prepared as follows: 100 μL of each sample was mixed with 400 μL of pre-chilled 80% methanol water solution, after which the resulting mixtures were vortexed, incubated in an ice bath for 5 min, and centrifuged at 4°C for 20 min at 15,000 × *g*. An appropriate volume of supernatant was diluted with mass spectrometry-grade water to the final methanol concentration of 53%, the samples were centrifuged again under the same conditions, and each supernatant was collected for mass spectrometry analysis. QC samples were prepared by mixing all experimental samples in equal volumes. Blank samples were treated with 53% methanol aqueous solution instead of serum and underwent the same treatment process.

Chromatographic separations were performed on an ultra-high performance liquid chromatography system equipped with a Hypersil Gold C18 column (Thermo Scientific) at 40°C and flow rate of 0.2 mL/min. Mobile phase A consisted of 0.1% formic acid in water, mobile phase B was methanol, and a gradient elution procedure was applied.

Mass spectrometry analysis was conducted using an ESI source in both positive and negative ion mode, with a scanning range of m/z 100–1500. The main parameters were as follows: spray voltage, 3.5 kV; sheath gas flow rate, 35 arb; auxiliary gas flow rate, 10 arb; ion-transport tube temperature, 320°C; and auxiliary gas heater temperature, 350°C. Secondary mass spectrometry fragmentation was performed in data-dependent acquisition mode.

The raw data were converted to mzXML format using ProteoWizard software, followed by peak extraction, peak alignment, and quantification using the XCMS program. Response correction was performed based on the first QC sample. Metabolite identification was performed by comparing the exact mass (mass deviation of 10 ppm) and secondary spectra against HMDB, LIPIDMaps, and other databases. After blank subtraction and normalization to the total peak area, compounds with a coefficient of variation of > 30% in the QC samples were removed, yielding the final quantitation matrix.

Multivariate statistical analysis, including principal component analysis and partial least squares-discriminant analysis, was performed using metaX software. Differential metabolites were identified based on variable importance in projection (VIP) > 1, a t-test P value of < 0.05, and an FC of ≥ 1.5 or ≤ 0.667. The KEGG database was used for pathway annotation and enrichment analysis. The R software packages ggplot2, pheatmap, and corrplot were used to draw volcano maps, clustering heat maps and correlation network maps, respectively. All calculations were carried out under the Linux operating system.

#### 2.5.7 Advanced oxidized protein product detection

The CheKine Advanced Oxidized Protein Product (AOPP) Content Detection Kit (micro method) was used for analysis. Freshly collected serum samples were diluted 1:4 with 1× extraction buffer. A standard curve (0–100 nmol/mL) was prepared via serial dilution using the standard master solution provided in the kit. We added 200 μL of diluted sample, standard, or buffer (blank) to different wells in a 96-well ultraviolet (UV)-transparent plate, after which 10 μL of Reagent I and 20 μL of Reagent II were added to each well. Following gentle mixing, the plate was incubated at 37°C for 5 min in a dark environment, then the absorbance (A) at 340 nm was immediately determined using a microplate reader. The ΔA was calculated for each well (ΔA = A assay – A blank). A standard curve was plotted with the standard concentration as the ordinate and ΔA as the abscissa, and a linear regression equation was obtained. The ΔA of each sample was substituted into the equation to determine the AOPP concentrations in the diluted samples, which were subsequently multiplied by the dilution factor (5) to obtain the AOPP contents in the original serum samples (nmol/mL).

#### 2.5.8 Protein carbonyl detection

PC levels in serum samples were determined using the CheKine Protein Carbonyl Content Detection Kit (micro method). The serum samples did not require pretreatment and were tested directly. In a 96-well UV-transparent plate, 60 μL of serum was added to each well. Then, 120 μL of chromogen solution was added to each well, an equal volume of HCl was added to the control well, and the plate was incubated for 1 h at 37°C. After the incubation, 150 μL of trichloroacetic acid was added to precipitate proteins, followed by centrifugation and removal of the supernatant. The pellet was washed thrice with anhydrous ethanol–ethyl acetate. The pellet was solubilized in 300 μL of guanidine hydrochloride and incubated at 37°C for 15 min. Next, the lysate was centrifuged, and the absorbance in the supernatant was determined at 370 nm. ΔA was calculated as A assay – A control, and the PC content was calculated according to the formula: PC content (μmol/mL) = 0.454 × ΔA × n (where n is the dilution factor).

#### 2.5.9 8-hydroxydeoxyguanosine detection

Competitive ELISA analysis was used for quantitative detection. Briefly, 50 μL of each standard or serum sample was added to a microplate pre-coated with an anti-8-OHdG antibody, followed by 100 μL of a horseradish peroxidase-labeled 8-OHdG antigen. After incubation at 37°C for 60 min, the plate was washed five times. Freshly prepared 3,3',5,5'-tetramethylbenzidine substrate solution was added, and the microplate was incubated at 37°C for 15 min, after which 50 μL of 2 M sulfuric acid was added to terminate the reaction. OD values were read at 450 nm. Sample OHdG concentrations were calculated by fitting a standard curve using a four-parameter logistic model. If the OD value of the sample exceeded the standard curve range, then the sample was appropriately diluted with the matching diluent and retested.

#### 2.5.10 Malondialdehyde detection

The Elabscience^®^ Malondialdehyde (MDA)Colorimetric Assay Kit (TBA Method) was used to detect MDA contents, per the manufacturer’s instructions. Blood samples were collected and centrifuged at 10,000 × *g* for 10 min at 4**°**C. Each supernatant was collected and kept on ice, with a portion set aside for subsequent protein concentration determination. The second and third working solutions of the reagent (50% acetic acid) and serial dilutions of the standards were prepared in advance. For the assay, 20 μL of each standard solution, test sample, and control sample were added to labeled 1.5 mL Eppendorf tubes. Next, 20 μL of Reagent 1 and 600 μL of Reagent 2 were sequentially added to each tube. Thereafter, 200 μL of Reagent 3 was added to the standard and sample tubes, whereas 200 μL of 50% acetic acid was added to the control tubes. The tube openings were sealed with plastic wrap, each wrap was punctured with a small hole after thorough mixing, and the tubes were incubated in a water bath at 100°C for 40 min. Following incubation, the tubes were cooled to room temperature under running water and centrifuged at 9,569 × *g* for 10 min. Finally, 250 μL of each supernatant was transferred to a microplate, and OD values were read at 532 nm.

#### 2.5.11 Superoxide dismutase detection

The Elabscience^®^ total Superoxide Dismutase (SOD)Activity Assay Kit (WST-1 method) was used to detect SOD contents, per the manufacturer’s instructions. Blood samples were collected and centrifuged at 10,000 × *g* for 10 min at 4°C. Each supernatant was collected and kept on ice, with a portion set aside for subsequent protein concentration determination. The enzyme working solution and substrate reaction solution were prepared in advance. For the assay, 20 μL of double-distilled water and 20 μL of enzyme working solution were added to the control wells. In the blank control wells, 20 μL of double-distilled water and 20 μL of reagent 4 were added. In the assay wells, 20 μL of the test sample and 20 μL of enzyme working solution were added. Subsequently, 200 μL of substrate solution was added to each well, followed by gentle mixing. The plates were incubated at 37°C for 20 min, and then OD values were read at 450 nm.

#### 2.5.12 Cell heat stroke model establishment and grouping

BV2 cells were used in the experiment. Cells were divided into four groups: control, 40 μM TBHQ, heat stroke (HS), and 40 μM TBHQ + HS. To establish the heat stroke model, cells in the logarithmic growth phase were placed in a 42 °C, 5% CO_2_ incubator for 2 hours, followed by transfer to a 37 °C, 5% CO_2_ incubator for recovery.

For drug pretreatment, the NRF2-specific agonist TBHQ was dissolved in DMSO and diluted with culture medium to a working concentration of 40 μM, ensuring the final DMSO concentration remained below 0.1%. TBHQ was added to the cell culture medium 24 hours prior to the induction of heat stroke.

#### 2.5.13 Western blotting experiment

##### 2.5.13.1 Protein extraction and quantification

Following treatment, cells were collected and lysed on ice for 30 minutes using RIPA lysis buffer containing PMSF. The lysates were centrifuged at 12,000 rpm for 15 min at 4 °C, and the supernatant containing total protein was collected. Protein concentration was determined using a bicinchoninic acid (BCA) assay. A standard curve was generated using serially diluted bovine serum albumin standards. Samples were diluted appropriately, mixed with the BCA working reagent, and incubated at 37 °C for 30 min. Absorbance was measured at 562 nm, and protein concentration was calculated based on the standard curve.

##### 2.5.13.2 Western blotting

Protein samples were mixed with 5× loading buffer, denatured by boiling at 100 °C for 10 min, and stored at -20 °C until use. SDS-PAGE gels were prepared according to the manufacturer’s instructions. Equal amounts of protein (20 μg per lane) were loaded and separated by electrophoresis. Electrophoresis was initially performed at a constant voltage of 80 V until the samples entered the resolving gel, then continued at 120 V until the dye front reached the bottom of the gel.

Proteins were transferred onto polyvinylidene difluoride (PVDF) membranes using a wet transfer system. The PVDF membrane was activated in methanol and assembled into a transfer stack in the order: cathode - sponge - filter paper - gel - PVDF membrane - filter paper - sponge - anode. Transfer was conducted at a constant current of 250 mA for 90 min with an ice bath to dissipate heat.

After transfer, the membrane was blocked with 5% skim milk at room temperature for 2 h with gentle agitation. The membrane was then washed three times with Tris-buffered saline containing Tween 20 (TBST), 5 min per wash. Subsequently, it was incubated with appropriately diluted primary antibodies overnight at 4 °C. Following primary antibody incubation, the membrane was washed three times with TBST and incubated with corresponding horseradish peroxidase (HRP)-conjugated secondary antibodies for 1 h at room temperature. After final washes, protein bands were visualized using an enhanced chemiluminescence (ECL) substrate. Band intensity was quantified using ImageJ software.

#### 2.5.14 Statistical methods

Statistical analyses were performed using GraphPad Prism (version 10.3.0) and Stata (version 20). For cell and animal experiments, continuous variables were first tested for normality and homogeneity of variance (Levene’s test). Data conforming to a normal distribution with homogeneity of variance were analyzed using repeated measures analysis of variance. Data with a normal distribution were expressed as the mean ± standard deviation, and differences between groups were assessed using one-way analysis of variance. A two-sided P-value of < 0.05 was considered to reflect a statistically significant difference. For patient data, continuous variables were also tested for normality. Variables with a normal distribution were expressed as the mean ± standard deviation, whereas those with a non-normal distribution were expressed as the median (interquartile range). Group comparisons for normally distributed variables were conducted using the independent-samples t-test, whereas non-normally distributed variables were analyzed with Mann-Whitney U tests. Categorical variables were analyzed using the chi-square test or Fisher’s exact test, as appropriate. A P-value < 0.05 was considered to represent a statistically significant difference.

## 3 Results

### 3.1 Summary of the patients’ clinical characteristics

**Figure 1:**
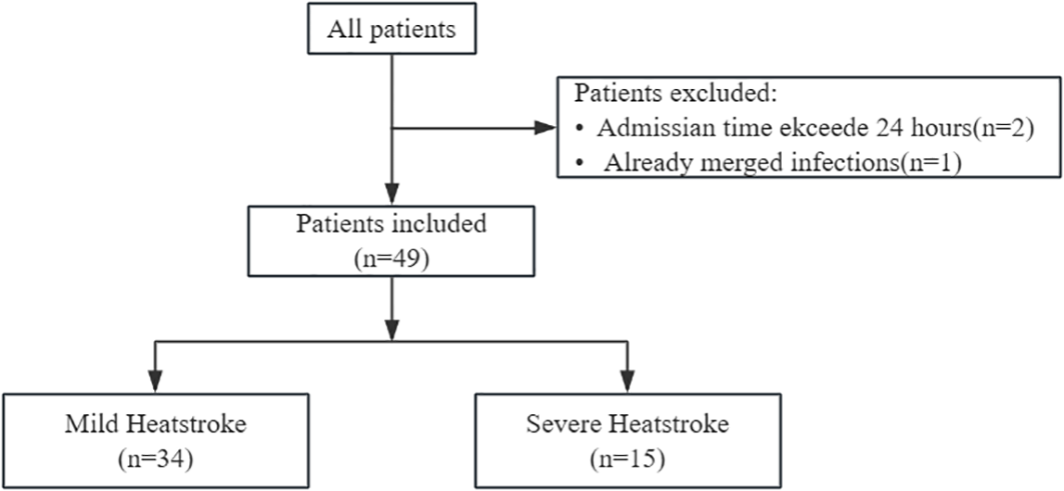
Patient enrollment and classification.

We included 49 patients with heat stroke in the study, comprising 34 patients with mild heat stroke and 15 patients with severe heat stroke (**Figure 1**). A total of 18 young males and 2 females were recruited as healthy controls, with a mean age of 25 years. The distribution of sex and age was matched with that of the heatstroke patients. The clinical characteristics of patients in the mild and severe groups are summarized in **Table 1**. Clinical data were collected from patients and classified into exertional heat stroke (EHS) and classic heat stroke (CHS). Among the 49 heatstroke patients, 42 were diagnosed with EHS, accounting for 86%. Specifically, 32 patients in the mild group were EHS, accounting for 94%, while 10 patients in the severe group were EHS, accounting for 67%. Compared with the mild heat stroke group, patients in the severe heat stroke group had significantly greater disease severity, higher levels of inflammation, and more pronounced organ dysfunction.

**Table 1:** Baseline characteristics by heatstroke**.

	Severity			
Variable	Overall (N = 49)	Mild heatstroke (N = 34)	Severe heatstroke (N = 15)	p-value
Age(year)	**24.00 (22.00, 27.00)**	**22.50 (22.00, 25.00)**	**27.00 (24.00, 47.00)**	**0.009**
EHS(%)	**42 (86%)**	**32 (94%)**	**10 (67%)**	**0.022**
Gender(%)	**46 (94%)**	**32 (94%)**	**14 (93%)**	**0.999**
HR(bpm)	**78.00 (60.00, 101.00)**	**72.50 (60.00, 88.00)**	**115.00 (58.00, 143.00)**	**0.018**
R(bpm)	**20.00 (18.00, 21.00)**	**20.00 (18.00, 20.00)**	**21.00 (19.00, 32.00)**	**0.031**
T(°C)	**38.40 (37.00, 39.40)**	**38.00 (36.70, 38.80)**	**39.20 (38.20, 40.20)**	**0.002**
Coma(%)	**14 (29)**	**4 (12)**	**10 (67)**	**<0.001**
Vasoactive drugs use(%)	**7 (14)**	**1 (2.9)**	**6 (40)**	**0.003**
Intubation(%)	**8 (16)**	**0 (0)**	**8 (53)**	**<0.001**
APACHE II	**6.00 (4.00, 15.00)**	**5.00 (3.00, 7.00)**	**20.00 (13.00, 24.00)**	**<0.001**
SOFA	**2.00 (1.00, 7.00)**	**1.00 (0.00, 3.00)**	**10.00 (6.00, 14.00)**	**<0.001**
WBC(10^9/L)	**10.73 (7.58, 13.64)**	**10.22 (7.34, 12.71)**	**12.53 (10.61, 17.91)**	**0.055**
CRP(mg/L)	**2.14 (0.69, 5.90)**	**2.41 (0.70, 8.44)**	**1.27 (0.55, 2.92)**	**0.437**
PCT(ng/ml)	**0.30 (0.05, 1.32)**	**0.08 (0.05, 0.23)**	**1.02 (0.30, 2.83)**	**0.002**
TBil(umol/L)	**18.10 (12.10, 29.00)**	**14.80 (11.65, 22.25)**	**29.20 (16.20, 60.90)**	**0.020**
Cre(μmol/L)	**128.50 (94.00, 158.00)**	**111.00 (87.00, 138.00)**	**154.00 (138.00, 209.00)**	**0.006**
CK(U/L)	**818.00 (277.00, 2,843.50)**	**480.00 (225.00, 1,339.00)**	**1,762.00 (681.00, 3,299.00)**	**0.032**
PT(sec)	**14.70 (13.90, 17.50)**	**14.45 (13.80, 16.10)**	**22.00 (15.00, 25.80)**	**0.001**
DD(mg/L)	**0.87 (0.22, 11.20)**	**0.37 (0.22, 0.64)**	**13.42 (5.94, 46.17)**	**<0.001**
PLT(10^9/L)	**185.00 (137.00, 234.00)**	**206.50 (177.00, 268.00)**	**59.00 (41.00, 155.00)**	**<0.001**
Time of hospital(day)	**6.00 (4.00, 10.00)**	**5.00 (3.00, 6.00)**	**11.00 (9.00, 56.00)**	**<0.001**
Outcome(%)	**2 (4.1)**	**0**	**2 (13)**	**0.164**

The bold numbers represent the median (interquartile range).

### 3.2 Multi-omics results

#### 3.2.1 Transcriptomics

**Figure 2:**
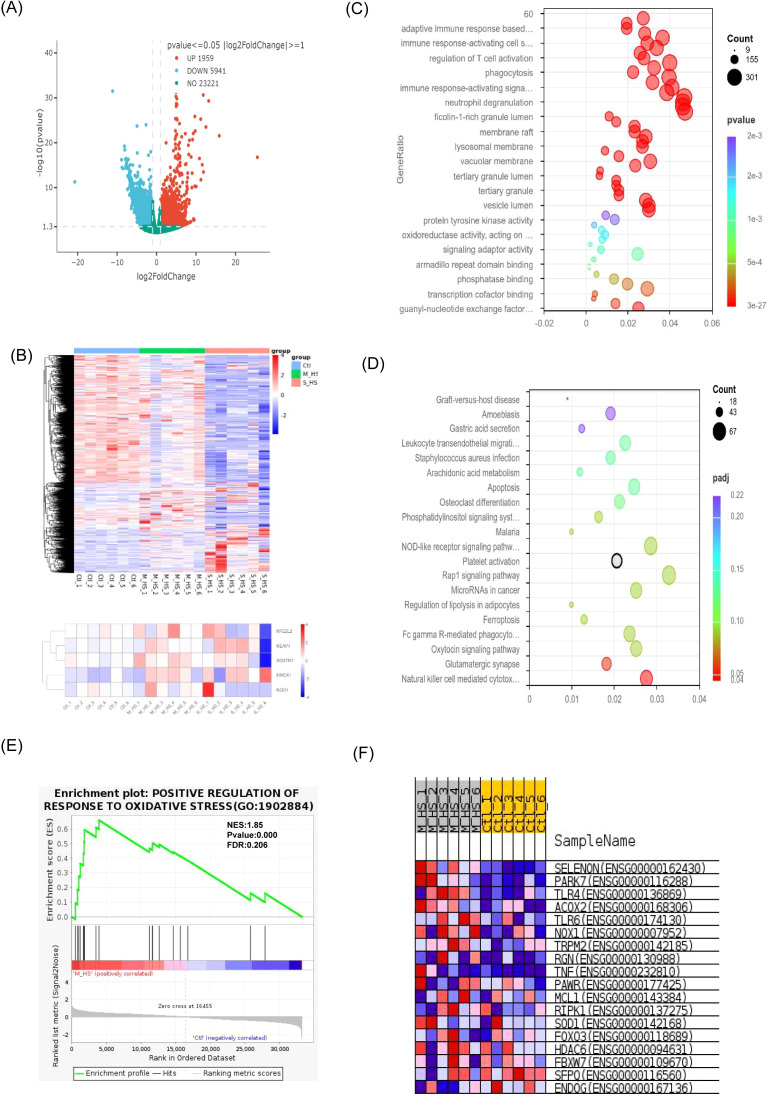
Transcriptional signatures in heat stroke patients are compared using RNA sequencing: **(A)** Genetic differential volcanogram (S_HS VS CTL); **(B)** Total cluster analysis heat map of differential genes; **(C)** KEGG analysis bubble diagram; **(D)** GO analysis bubble chart; **(E)** GSEA analysis; **(F)** Significant enrichment of gene sets that positively regulate oxidative stress. GO, gene ontology; GSEA, gene set enrichment analysis; KEGG, Kyoto Encyclopedia of Genes and Genomes.

Transcriptional profiles of patients with heat stroke were analyzed via RNA sequencing. Patients with severe heat stroke exhibited markedly different transcriptional patterns than did healthy controls. Differentially expressed genes were identified using the criteria, |log_2_ (FC)| ≥ 1 and p ≤ 0.05. Significant expression differences were observed in both the mild and severe heat stroke groups, when compared with the expression profile of the healthy control group. We identified 7,900 differentially expressed genes between the severe heat stroke and healthy control groups, including 1,959 upregulated genes and 5,941 downregulated genes (**Figure 2A**). Cluster analysis of differentially expressed genes demonstrated substantial changes in the NRF2–HO-1 antioxidant pathway following heat stroke. *NFE2L2*, *KEAP1*, *SQSTM1*, *HMOX1*, and *NQO1* showed significantly higher expression in patients with mild heat stroke than in the control group. In contrast, in patients with severe heat stroke, *KEAP1*, *SQSTM1*, and *HMOX1* remained considerable upregulated, whereas *NFE2L2* and *NQO1* were significantly downregulated, when compared with the corresponding expression levels in the control and mild groups (**Figure 2B**). KEGG and GO enrichment analyses of these differentially expressed genes revealed significant enrichment in pathways related to inflammatory immune activation, natural killer cell-mediated cytotoxicity, and apoptosis (**Figures 2C, D**). GSEA, comparing normal controls and patients with mild heat stroke, showed significant enrichment of gene sets involved in positively regulating oxidative stress, such as *SELENON*, *PARK7*, *TLR4*, *ACOX2*, *TLR6*, *NOX1*, and *TRPM2* (**Figures 2E, F**).

#### 3.2.2 Proteomics

**Figure 3:**
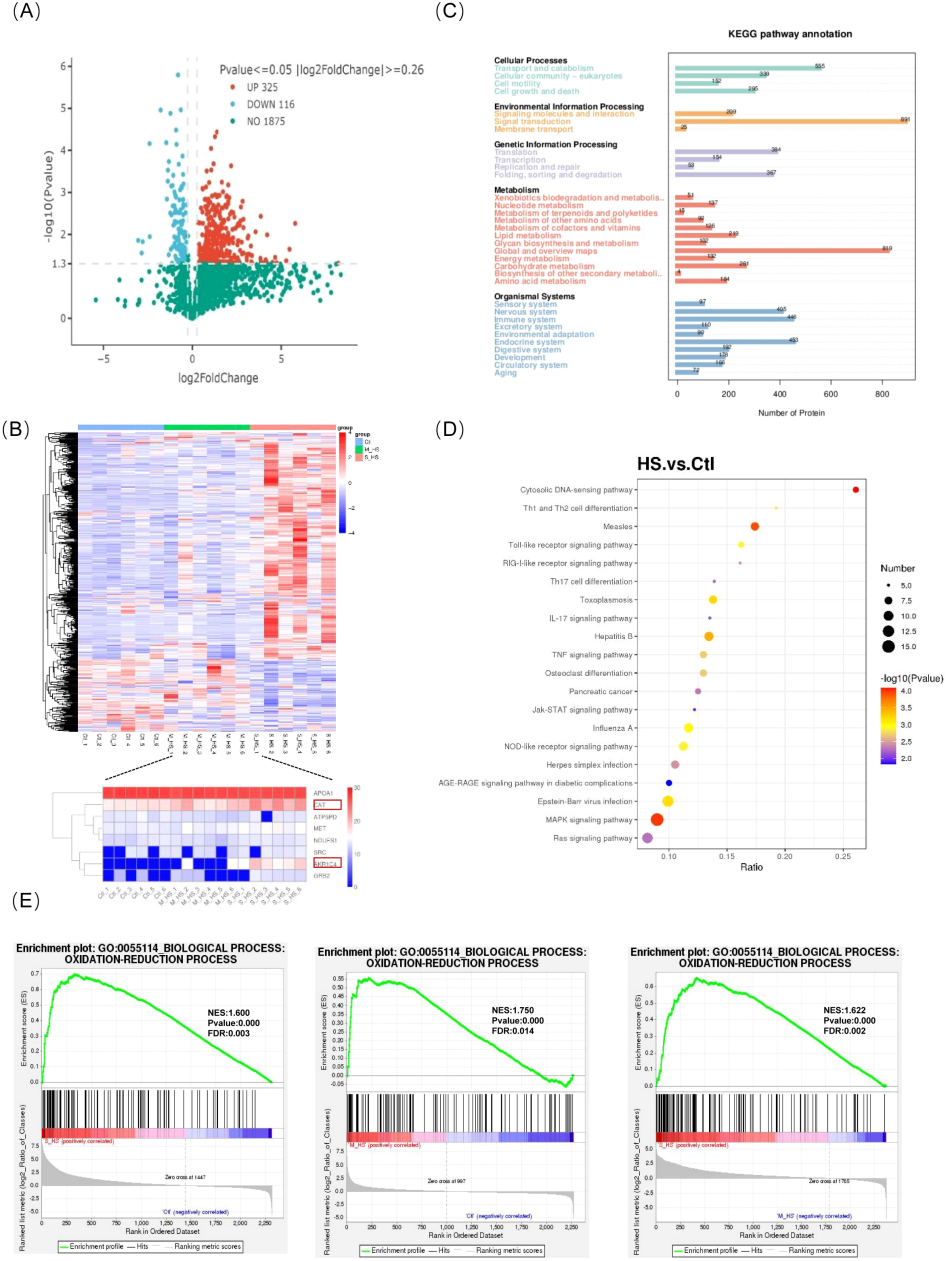
Proteomics: **(A)** Protein differential volcanogram; **(B)** Differential protein total cluster analysis heat map, **(C)** KEGG pathway enrichment; **(D)** KEGG enrichment bubble diagram; **(E)** GSEA-GO ES is enriched, and the gene set that positively regulates redox processes after heat stroke is significantly enriched. GO, gene ontology; GSEA, gene set enrichment analysis; KEGG, Kyoto Encyclopedia of Genes and Genomes.

Differentially expressed proteins were identified using the criteria of FC > 1.2 and p < 0.05 for upregulation and FC < 0.83 and p < 0.05 for downregulation. When comparing the severe heat stroke and control groups, we found that 465 proteins were differentially expressed, including 347 upregulated and 118 downregulated proteins (**Figure 3A**). Hierarchical clustering analysis demonstrated different protein-expression patterns in patients with heat stroke than in healthy controls. Notably, the levels of antioxidant enzymes such as catalase and aldo-keto reductase family 1 member C4 were significantly altered following heat stroke (**Figure 3B**). KEGG enrichment analysis revealed that these differentially expressed proteins were significantly enriched in pathways related to oxidative phosphorylation, tumor necrosis factor signaling, and IL-17 signaling, among other pathways (**Figures 3C, D**). GSEA further showed significant enrichment of gene sets that positively regulate redox processes in patients with heat stroke (**Figure 3E**).

#### 3.2.3 Metabolomics

**Figure 4:**
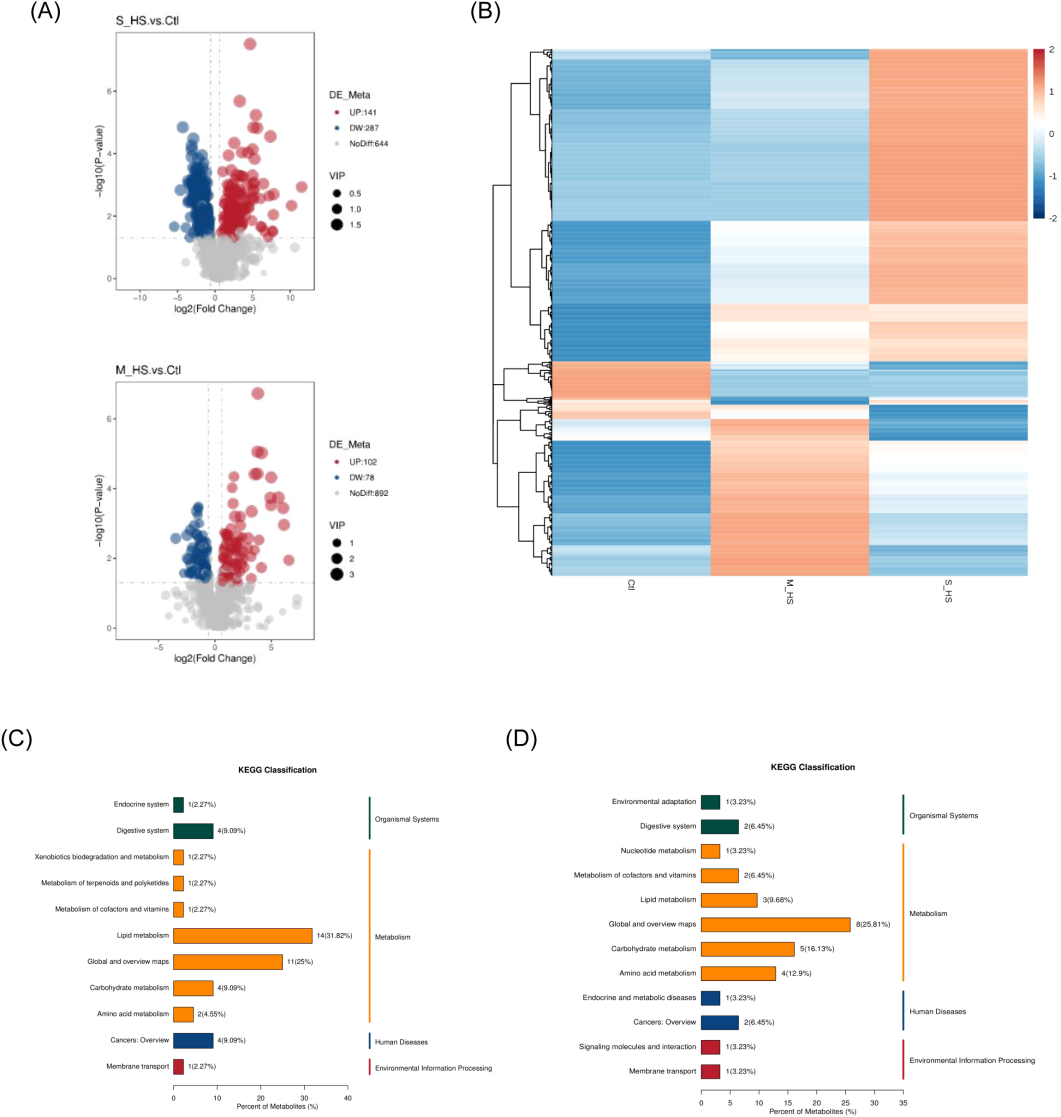
Non-targeted metabolomics analysis: **(A)** Differential metabolite cluster volcano map (S_HS VS CTL) (M_HS VS CTL); **(B)** Differential metabolite cluster volcanic map; **(C)** KEGG Enrichment Analysis (S_HS VS Ctl) **(D)** KEGG Enrichment Analysis (M_HS VS Ctl). KEGG, Kyoto Encyclopedia of Genes and Genomes.

Differential metabolites were identified using the thresholds, VIP > 1.0, FC > 1.5 or FC < 0.667, and p < 0.05. Both negative mode- and positive-mode acquisitions were applied. Clustering heatmaps and volcano plots revealed numerous metabolites that were significantly altered between patients with heat stroke and healthy controls. When comparing the mild heat stroke and control groups, we observed that 560 metabolites were differentially expressed, including 434 upregulated and 126 downregulated metabolites. In the severe heat stroke vs control comparison, 914 metabolites were differentially expressed, of which 560 were upregulated and 354 were downregulated (**Figures 4A, B**). KEGG enrichment analysis of these differentially produced metabolites showed that they were primarily associated with pathways in the nervous and digestive systems. Regarding metabolic functions, they were significantly enriched in terms of lipid metabolism, carbohydrate metabolism, and amino acid metabolism (**Figures 4C, D**).

#### 3.2.4 Joint multi-omics analysis

**Figure 5:**
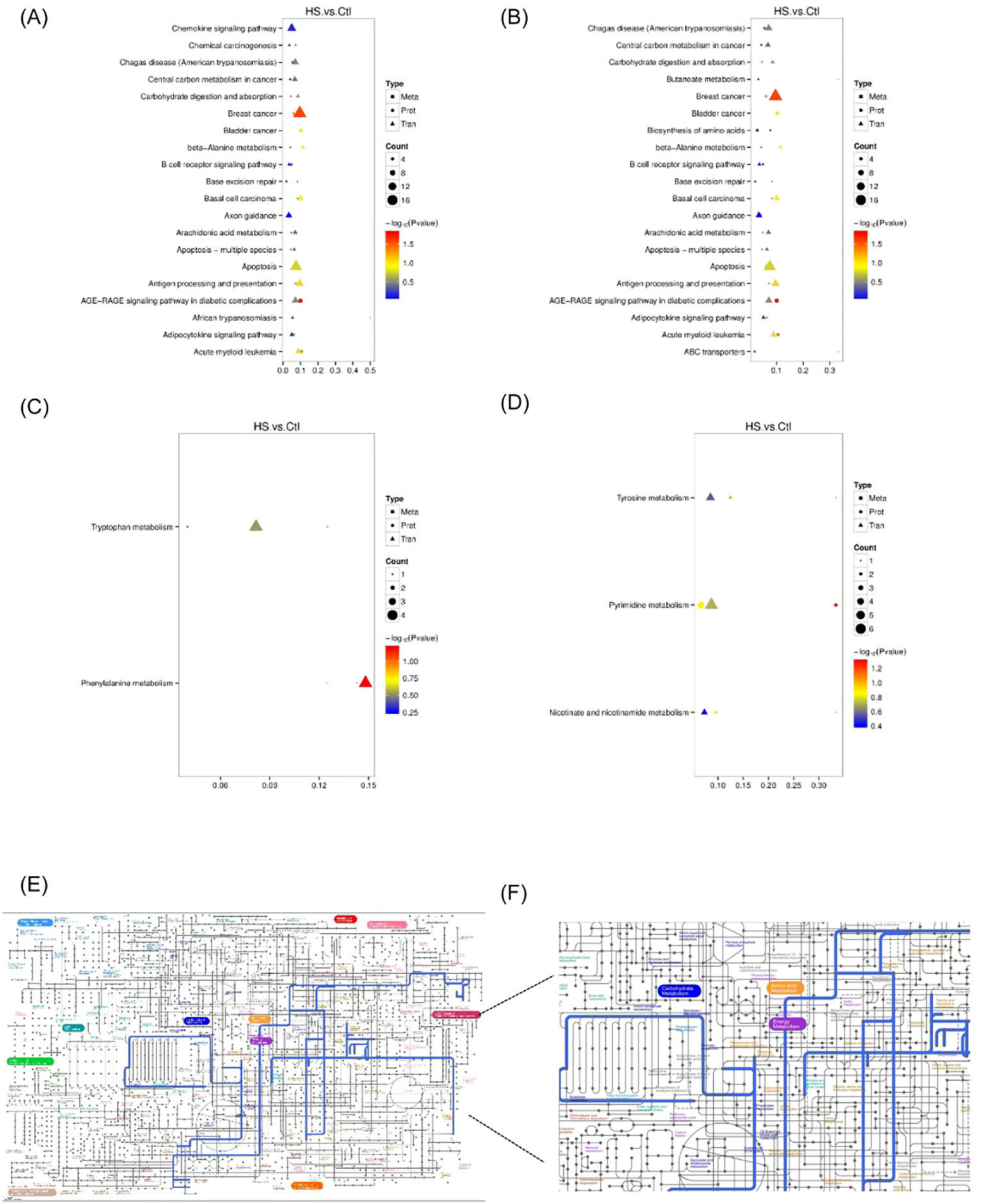
Combined analysis of transcriptomics, proteomics and non-targeted metabolomics: KEGG enrichment bubble diagram of differential genes, differential proteins and differential metabolites **(A)** positive ion mode and **(B)** negative ion mode; Differential genes, differential proteins and differential metabolites KEGG enrichment bubble diagram **(C)** positive ion mode, **(D)** negative ion mode; **(E, F)** iPath pathway map of differential genes, differential proteins, and differential metabolites. KEGG, Kyoto Encyclopedia of Genes and Genomes.

All differentially expressed genes, proteins, and metabolites were mapped to the KEGG pathway database to identify shared pathway information. This analysis revealed the major biochemical and signal transduction pathways involving these differential molecules, with selected results presented below. The pathways shared by transcriptomics and proteomics were mainly associated with apoptosis (**Figures 5A, B**). Joint analysis of the three omics layers identified tryptophan metabolism and phenylalanine metabolism in positive-mode acquisition (**Figure 5C**), as well as tyrosine metabolism, pyrimidine metabolism, and niacin and nicotinamide metabolism in negative-mode acquisition (**Figure 5D**). KEGG enrichment analysis suggested that heatstroke was related to oxidative stress pathways to a certain extent (**Supplementary Material S1** for details). Furthermore, simultaneous mapping of differential metabolites and genes to the iPath database suggested that carbohydrate metabolism, amino acid metabolism, and mitochondrial energy metabolism may represent common pathways (**Figures 5E, F**).

### 3.3 Antioxidant *NRF2* expression, oxidative stress, and inflammation levels

**Figure 6:**
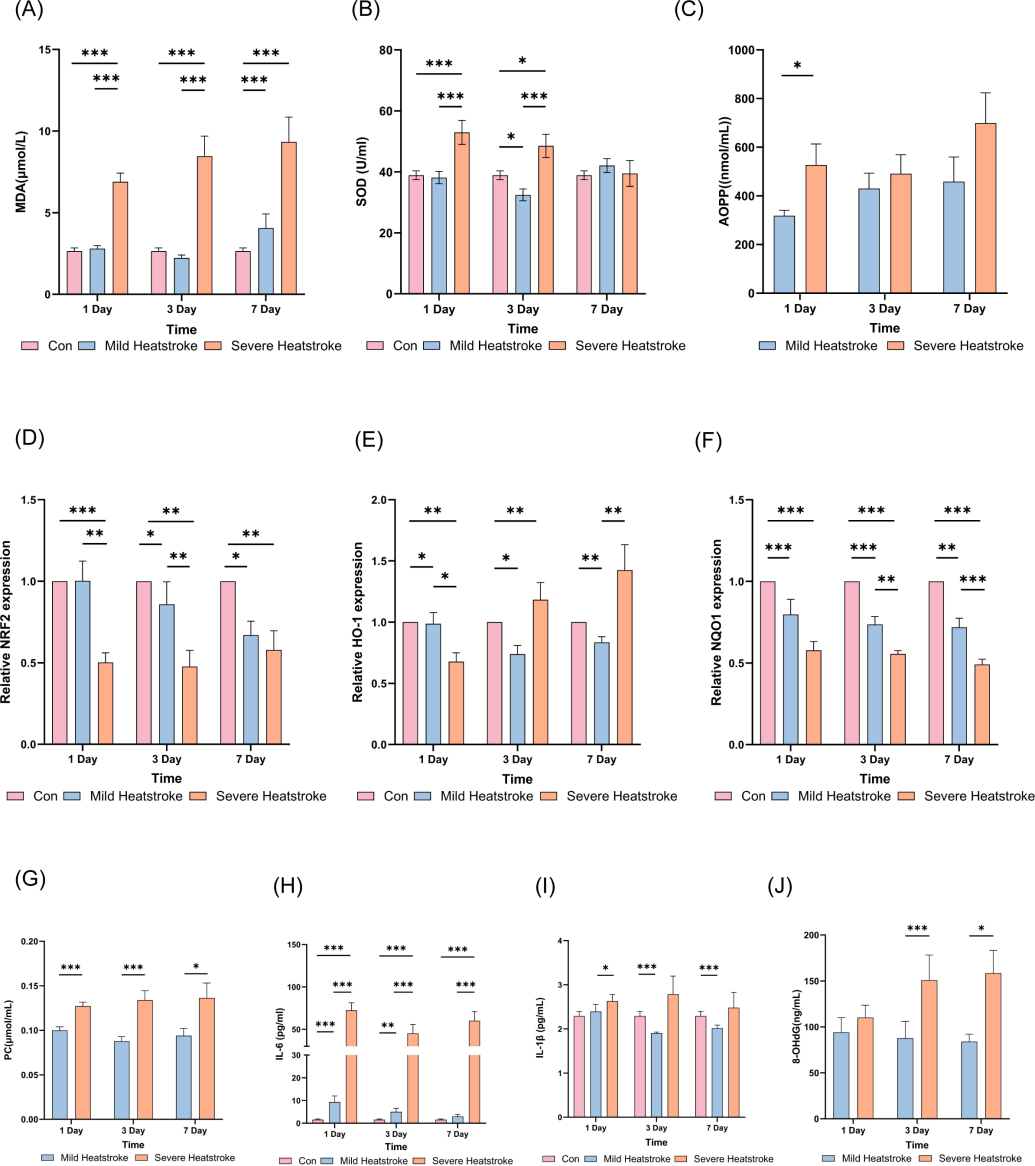
*NRF2* antioxidant gene, oxidative stress and inflammatory expression in patients with mild and severe heat stroke **(A)** MDA test results; **(B)** SOD level; **(C)** AOPP test results; **(D)** *NRF2* mRNA expression level; **(E)** *HO-1* mRNA expression level; **(F)** *NQO1* mRNA expression level.; **(G)** PC test results; **(H)** IL-6 test results; **(I)** *IL-1β* mRNA expression level; **(J)** 8-OHdG test results.MDA, malondialdehyde; SOD, superoxide dismutasse; AOPP, Advanced Oxidized Protein Product; PC, Protein Carbonyl; 8-OhdG, 8-Hydroxydeoxyguanosine.

In patients with severe heat stroke, MDA levels were significantly elevated and showed a trend of gradually increasing with the progression of the disease (**Figure 6A**). Superoxide dismutase activity increased significantly on the first day of severe heat stroke but declined progressively as the disease advanced (**Figure 6B**). To further investigate the oxidative stress levels of patients with heat stroke, we tested the content of advanced protein oxidation products (AOPP), protein carbonyl (PC), and 8-hydroxydeoxyguanosine (8-OHdG). The AOPP level of patients with severe heat stroke was significantly higher than that of patients with mild heat stroke on the first day, decreased slightly on the third day (becoming close to that of patients with mild heat stroke), and increased again on the seventh day (**Figure 6C**). The trend of changes in PC and 8-OHdG levels was consistent with that of MDA (**Figures 6G, J**). Compared with the healthy control and mild heat stroke groups, expression of the *NFE2L2* (*NRF2*) gene was significantly reduced in patients with severe heat stroke on days 1 and 3, and remained lower than controls on day 7, although not significantly different from mild cases. The expression of *NFE2L2* was also decreased in mild heat stroke compared with controls (**Figure 6D**). The expression of the *HMOX1* (HO-1) gene was significantly reduced in mild heat stroke on days 3 and 7. In patients with severe heat stroke, *HMOX1* expression initially decreased on day 1 but then increased significantly on days 3 and 7 compared with both the control and mild groups (**Figure 6E**). The expression of the *NQO1* gene was reduced in all patients after heat stroke, with significantly lower levels observed in severe cases compared with both the control and mild groups (**Figure 6F**). IL-6 levels were significantly elevated in severe cases, whereas IL-1β levels showed no significant change (**Figures 6H, I**). Furthermore, we separately analyzed the relationships between the levels of SOD, MDA, AOPP, PC, 8-OHdG, and NRF2 in patients with mild heat stroke and patients with severe heat stroke, respectively. The corresponding results are provided in **Supplementary Material S2**. The results indicate that in patients with mild heat stroke, the levels of SOD and NRF2 show a consistent trend, both increasing with disease progression, which suggests a relatively robust antioxidant capacity in this group. Conversely, in patients with severe heat stroke, SOD and NRF2 levels also exhibit a concordant trend, but characterized by a decrease with disease progression, indicating that the antioxidant capacity is indeed compromised in patients with severe heat stroke.

### 3.4 Trend in multiple organ function damage

**Figure 7:**
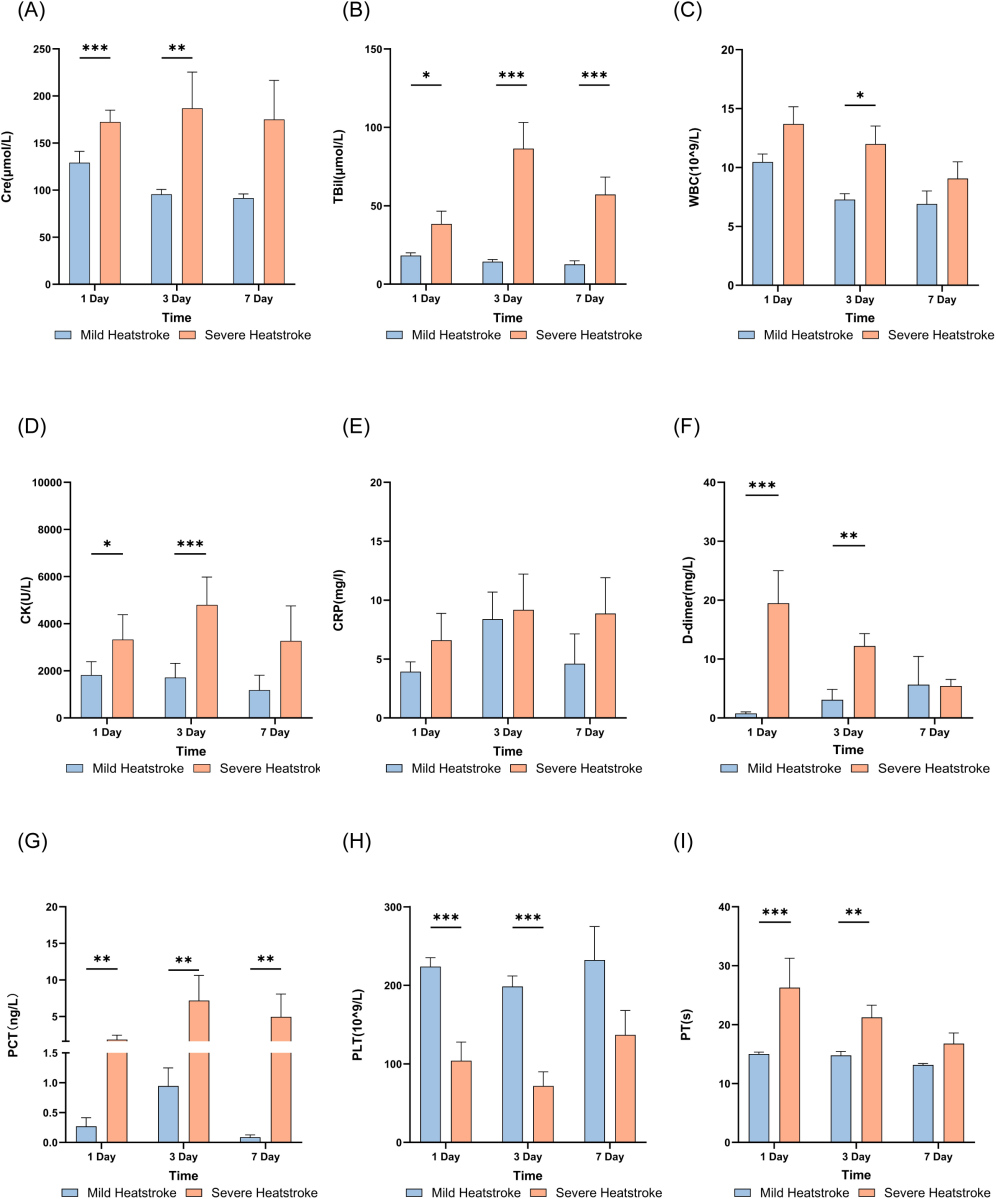
Trend of multi-organ functional damage in patients with heat stroke **(A)** Cre results; **(B)** TBil results; **(C)** WBC count; **(D)** CK results; **(E)** CRP results; **(F)** D-dimer results; **(G)** PCT results; **(H)** PLT results; **(I)** PT results. Cre,creatinine;TBil, total bilirubin;WBC,white blood cell count;CK,creatine kinase;CRP,C-reactive protein;PCT,procalcitonin;PLT,platelet count; PT, prothrombin time.

Patients with severe heat stroke exhibited more pronounced pathological changes, including multi-organ injury and rhabdomyolysis, than did those with mild heat stroke (p < 0.05). Serum creatinine levels were significantly higher in severe cases of heat stroke than in mild cases and continued to rise with disease progression, whereas levels in mild cases declined over time (**Figure 7A**). Total bilirubin levels in severe cases were also significantly higher than in mild cases, increasing further from day 1 to 3 and remaining elevated on day 7 (**Figure 7B**). White blood cell counts were higher in severe cases than in mild cases, with values gradually decreasing in both groups as the disease progressed (**Figure 7C**). Creatine kinase levels were significantly higher in severe cases on day 3 than in mild cases but returned to near day 1 levels by day 7 (**Figure 7D**). C-reactive protein levels in patients with severe heat stroke were slightly higher than in patients with mild heatstroke, but this difference was not significant (**Figure 7E**). D-dimer production was markedly elevated in patients with severe heat stroke on day 1 (**Figure 7F**). Procalcitonin levels were significantly higher in severe cases than in mild cases, reaching a peak before subsequently declining (**Figure 7G**). Platelet counts were significantly lower in severe cases on days 1 and 3 than in mild cases but showed recovery by day 7 (**Figure 7H**). The prothrombin time was significantly longer in the severe heat stroke group than in the mild heat stroke group but gradually shortened with clinical improvement (**Figure 7I**).

### 3.5 Correlation between *NFE2L2* expression and the SOFA score

**Figure 8:**
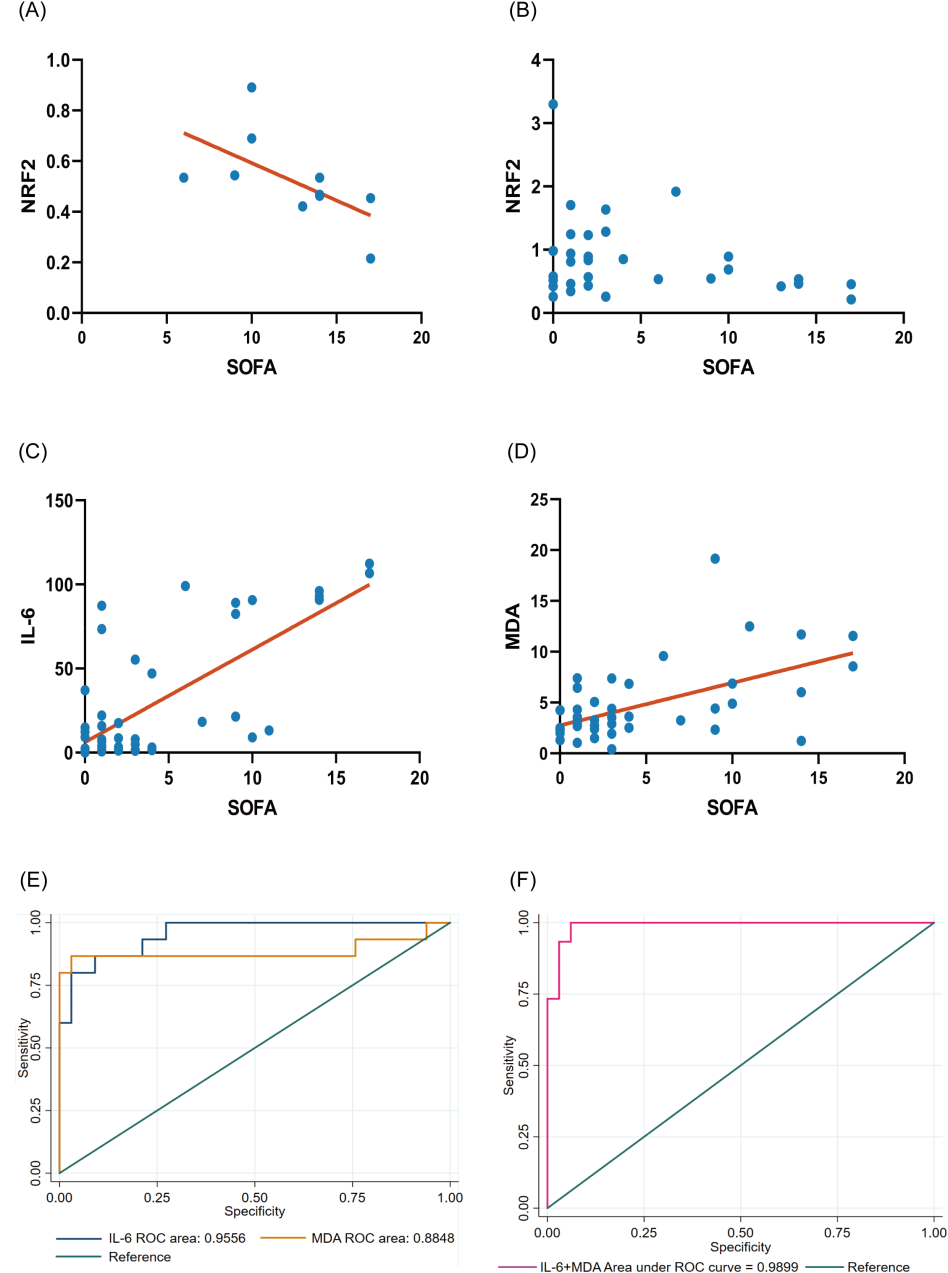
Correlation between SOFA score NFE2L2 (NRF2) gene and SOFA score **(A)** In none of the patients with heat stroke, NFE2L2 (*NRF2*) expression is significantly correlated with SOFA or APACHE II scores. **(B)** Subgroup analysis of patients with severe heat stroke shows a significant positive correlation between *NFE2L2* expression and SOFA score; **(C)** *IL-6* levels are strongly positively correlated with SOFA scores. **(D)** *MDA* levels are also significantly positively correlated with SOFA scores. **(E)** the respective relationship between IL-6 and MDA and SOFA score; **(F)** Relationship between IL-6 and MDA combined use with SOFA scores. APACHE, Acute Physiology and Chronic Health Evaluation; MDA, malondialdehyde; SOFA, Sequential Organ Failure Assessment.

Across all patients with heat stroke, *NFE2L2* (NRF2) expression did not correlate with either SOFA or APACHE II scores (**Figure 8B**). However, subgroup analysis of patients with severe heat stroke revealed a significant positive correlation between *NFE2L2* expression and SOFA scores (**Figure 8A**). In addition, IL-6 levels correlated strongly and positively with SOFA scores (R^2^ = 0.5237, p < 0.0001; **Figure 8C**), and MDA levels correlated positively with SOFA scores (R^2^ = 0.3282, p < 0.0001; **Figure 8D**). Combined analysis demonstrated that using IL-6 and MDA together improved the predictive performance for heat stroke severity, with the area under the curve increasing from 0.8848 for MDA alone and 0.9556 for IL-6 alone to 0.9899 when the two markers were combined (**Figures 8E, F**).

### 3.6 *In vitro* experiments preliminarily confirmed the regulatory role of NRF2 in oxidative stress and inflammation

*In vitro* experiments showed that the NRF2 expression increased significantly after adding TBHQ compared with the heat stroke group alone, and the HO-1 expression level showed a consistent trend with NRF2. Compared with the heat stroke group alone, the levels of NLRP3 and IL1β decreased after NRF2 activation, suggesting that NRF2 could reduce inflammation by regulating oxidative stress (**Supplementary Figure 3**). Yin et al. demonstrated that pretreatment with TBHQ activates the NRF2 signaling pathway and attenuates heat stroke-induced injury (7).

## 4 Discussion

The overall mortality rate of severe heat stroke remains high, and the extent of multi-organ dysfunction strongly influences patient prognosis (8). Current clinical treatment options are limited, with anti-inflammatory therapy representing a primary approach (1). In addition to the direct inflammatory damage activated by primary fever, basic research has shown that endothelial damage, mitochondrial dysfunction, and oxidative stress are important factors that aggravate inflammatory activation (9). One of our research goals is to deepen the study of oxidative stress-related pathways through multi-omics techniques, providing a basis for the future expansion of new antioxidant and anti-inflammatory adjuvant therapies. Presently, clinical research on oxidative stress injury and antioxidant defense mechanisms remains limited (2). Our literature review identified 30 studies related omics studies, of which only six were designed as clinical studies for patients with heat stroke (10–16).

In this study, GSEA was performed across transcriptomic, proteomic, and metabolomic datasets. Gene expression related to oxidative stress was significantly elevated after heat stroke, indicating that oxidative stress dysregulation contributes to heat stroke-associated injury. This result is consistent with findings reported by Yin et al. and Abderrezak Bouchama et al., suggesting that oxidative stress damage is crucial in heat stroke (10, 16). Further genetic screening revealed abnormal expression of *NFE2L2* (NRF2)-related genes. In the mild heat stroke group, the expression of *NFE2L2* and its downstream gene *HMOX1* (HO-1) was significantly elevated, whereas both were markedly decreased in the severe heat stroke group. These findings suggest that NRF2–HO-1-mediated antioxidant activity is enhanced in mild heat stroke but becomes insufficient in severe cases, resulting in inadequate antioxidant protection. These results are generally consistent with the data reported by Abderrezak Bouchama et al. (16); however, they also found that female patients with heat stroke exhibited greater oxidative stress damage than did males and higher NRF2 activation, although those findings have not been experimentally verified in subsequent studies.

Proteomic analysis further identified abnormal expression of the antioxidant proteins catalase and aldo-keto reductase family 1, member C4. Unlike the transcriptomic results, catalase protein expression progressively increased from mild to severe heat stroke, indicative of stronger antioxidant activity in the severe group. In contrast, aldo-keto reductase family 1 member C4 expression was low in both healthy controls and patients with mild heat stroke, but catalase levels rose significantly in severe cases. Metabolomic KEGG enrichment analysis showed that differential metabolites were primarily enriched in the nervous and digestive systems at the organ-system level, as well as in lipid, carbohydrate, and amino acid metabolism at the metabolic level. These findings indicate that heat stroke disrupted multiple biological systems and metabolic pathways, with the nervous system representing a major target of metabolic dysregulation.

Integrated multi-omics analysis revealed that transcriptomic, proteomic, and metabolomic changes were collectively enriched for tryptophan, phenylalanine, and tyrosine metabolism pathways. These pathways are closely linked to immune regulation, inflammatory responses, and energy metabolism. Notably, tryptophan metabolism plays a key role in modulating oxidative stress (17), further supporting the involvement of oxidative stress in the pathogenesis of heat stroke.

Previous findings by Du et al. and Wang et al. showed that heat stress (HS)-induced acute lung injury (ALI) is accompanied by the accumulation of reactive oxygen species (ROS), malondialdehyde (MDA), and Fe^2+^, along with the depletion of glutathione (GSH). Activation of the Nrf2 pathway can transcriptionally upregulate the expression of target genes such as NQO1, HO-1, GPX4, and SLC7A11, thereby correcting this redox imbalance and ultimately inhibiting (or blocking) the occurrence of ferroptosis (18, 19).Unlike the above-mentioned studies, we studied NRF2 in patients with clinical heat stroke for the first time in this study. Subsequent validation showed that, compared with both the healthy control and mild heat stroke groups, the severe heat stroke group had markedly lower *NFE2L2* (NRF2) expression levels on days 1 and 3, which remained lower than those in controls on day 7 but did not differ significantly from those in the mild heat stroke group. Overall, NRF2 expression was consistently suppressed in the severe heat stoke group. Patients with mild heat stroke also demonstrated lower *NFE2L2* expression than did controls. These core results also validate the results of our omics and previous animal studies, suggesting that NRF2 expression in the blood of patients with severe heat stroke decreased, in turn meaning that the corresponding antioxidant capacity of patients with severe heat stroke was insufficient.

Similar patterns were observed for the downstream phase II detoxification enzyme genes *HMOX1* (HO-1) and *NQO1*, suggesting that NRF2-mediated antioxidant activity was impaired. *HMOX1* expression in mild heat stroke was significantly reduced on days 3 and 7, whereas in severe heat stroke it showed a biphasic pattern—decreasing on day 1 but increasing significantly on days 3 and 7 compared with that in both control and mild heat stroke groups. *NQO1* expression was significantly lower in the severe heat stroke group than in the control and mild heat stroke groups. Biochemical assays supported these transcriptomic findings. Compared to patients with mild heat stroke, patients with severe heat stroke exhibited significantly higher levels of oxidative damage markers, including malondialdehyde (MDA), protein carbonyl (PC), and 8-hydroxy-2′-deoxyguanosine (8-OHdG), which showed a continuous increasing trend with disease progression. In patients with severe heat stroke, the level of advanced oxidation protein products (AOPP) was significantly higher than that in the mild heat stroke group on the first day of onset, briefly decreased to a level close to that of the mild group on day 3, and increased again on day 7. These changes suggest that reduced antioxidant capacity may further exacerbate oxidative stress damage. Furthermore, superoxide dismutase (SOD) activity in patients with severe heat stroke was higher than that in patients with mild heat stroke at the early stage (day 1), but subsequently declined rapidly, indicating that their endogenous antioxidant defense system was insufficient to effectively counteract the persistently heightened oxidative stress.

Although several preclinical findings have demonstrated the benefits of antioxidant therapy in sepsis and heat stroke, its protective effects in clinical settings remain inconclusive (20, 21). The NRF2-mediated antioxidant pathway represents one of the most important defense mechanisms against oxidative stress (22). The protective role of NRF2 has been repeatedly confirmed in preclinical studies of multiple critical illnesses (6). However, research specifically addressing the NRF2 antioxidant pathway in clinical studies of heat stroke remains lacking.

We performed statistical analysis to explore the relationships between NRF2, oxidative stress, inflammation, and disease severity to further explore these relationships in patient with heat stroke. In addition, we preliminarily demonstrated that enhanced NRF2 expression can simultaneously reduce oxidative stress and inflammatory damage in an *in vitro* heat stroke model (**Supplementary Material S2**). Across all patients with heat stroke, *NFE2L2* (NRF2) expression did not show significant correlations with SOFA or APACHE II scores. However, subgroup analysis of the severe heat stroke group demonstrated a clear positive correlation between *NFE2L2* expression and SOFA scores, suggesting that reduced NRF2 expression is more closely linked to disease progression in severe cases.

In mild heat stroke, oxidative stress and tissue injury are generally insufficient to produce marked systemic dysfunction, whereas cases of severe heat stroke (similar to sepsis) are characterized by profound decompensation of inflammatory, oxidative stress, and coagulation pathways. The stronger association of NRF2 with SOFA rather than APACHE II may reflect the fact that SOFA emphasizes multi-organ dysfunction, which represents the central clinical manifestation of heat stroke. The results of several retrospective studies, including one of our own, showed that the SOFA score provides more reliable prognostic value for heat stroke than does the APACHE II score (23–25).

In severe illness, particularly sepsis, oxidative stress and inflammatory activation interact closely, and assessing their combined state may provide a more accurate reflection of overall disease severity (17, 26). Correlation analysis in our cohort revealed significant positive associations between both MDA and IL-6 with SOFA scores in patients with heat stroke. Receiver operating characteristic curve analysis further demonstrated that combining IL-6 with MDA improved predictive accuracy in severe heat stroke compared with that of IL-6 alone, indicating that integrating markers of oxidative stress with inflammatory mediators better reflects the pathogenic mechanisms underlying severe heat stroke in addition to inflammation itself.

We also examined gene expression, inflammation, oxidative stress, and multi-organ function in patients with heat stroke on days 1, 3, and 7 after onset (**Supplementary Material S4** for details). Along with the correction of disseminated intravascular coagulation and decline of inflammatory markers—paralleled by the gradual recovery of liver and kidney function—*NFE2L2* (NRF2), *HMOX1* (HO-1), and *NQO1* expression showed upward trends. Therefore, NRF2-mediated antioxidant capacity progressively recovered as the disease improved. This observation indicates that the NRF2-controlled pathway correlated with disease severity after heat stroke and that NRF2-mediated antioxidant activity may contribute to limiting both oxidative and inflammatory damage. Despite these improvements, MDA and IL-6 levels remained elevated for 7 d after the heat stroke. This persistence implies that early antioxidant therapy may help mitigate oxidative stress injury and the heightened inflammatory response. In sepsis, numerous studies were designed to investigate early antioxidant therapy, with many focusing on the interplay between oxidative stress and inflammation (27). Preclinical data have confirmed that antioxidant interventions can attenuate both oxidative and inflammatory injury. However, clinical evidence remains limited. Some clinical reports showed negative results, although many were constrained by outcome measures, potentially overlooking beneficial auxiliary effects that, while not decisive, may still play important roles (28). For the first time, we explored NRF2-related antioxidant genes in patients with heat stroke and described the temporal expression patterns of *NFE2L2* and downstream genes, as well as their relationships with oxidative stress, inflammation, and organ function.

This study has several limitations. First, heat stroke is relatively rare in clinical practice, and this single-center study had a limited severe subgroup (n=15), restricting statistical power and generalizability. Due to the limited sample size, longitudinal data exhibited a non-normal distribution, and the residuals did not satisfy the assumption of normality; consequently, linear mixed-effects models and other parametric repeated-measures statistical methods were considered unsuitable. We instead performed the Kruskal-Wallis test with Bonferroni correction for data analysis; however, this approach did not fully account for within-subject correlations, and effective subgroup analyses (e.g., stratified by sex, age, or heat stroke type) were also precluded. Future studies should expand the cohort, employ more appropriate repeated-measures statistical models when assumptions are met, and incorporate independent replication cohorts for external validation. Second, the small sample size precluded effective modeling of confounders (e.g., comorbidities, medications, prehospital cooling) and assessment of their impact on the results, as well as robust prognostic model fitting to systematically evaluate the predictive value of the NRF2 axis for adverse outcomes such as organ failure or mortality. Third, proteomics and metabolomics analyses did not include multiple-testing correction for differentially expressed features, which may increase the risk of false positives; given that these omics data served auxiliary roles and the number of significant hits was limited after fold-change filtering, we opted not to perform additional correction, though stricter methods should be considered in future validation studies. In future investigations, the range of oxidative stress markers, antioxidant proteins, and inflammation-related molecules assessed should be expanded to provide a more comprehensive understanding of NRF2-related antioxidant effects. In addition, antioxidant drugs should be evaluated clinically to better clarify their therapeutic potential in heat stroke.

In conclusion, the results of this prospective study demonstrate that severe heat stroke was characterized by marked suppression of *NFE2L2* (NRF2) expression and its downstream antioxidant genes, accompanied by elevated oxidative stress, inflammatory activation, and multi-organ dysfunction. The positive correlation between NRF2 expression and SOFA scores in severe cases highlights the potential role of NRF2 as a biomarker of disease progression. Our findings suggest that targeting NRF2-mediated antioxidant pathways, particularly during the early stages of severe heat stroke, may represent a promising direction for therapeutic intervention.

## Data Availability

The datasets presented in this study can be found in online repositories. The names of the repository/repositories and accession number(s) can be found in the article/**Supplementary Material**.
