# Supplementary material for: Nuclear factor erythroid 2-related factor 2 gene expression in patients with heat stroke and its association with oxidative stress and inflammation: a prospective study
Source: Front Immunol. 2026 Apr 16;17:1719289. doi: 10.3389/fimmu.2026.1719289 (PMC13128558; doi:10.3389/fimmu.2026.1719289)
Supplement: Supplementary file 1 [file DataSheet1.pdf]

Supplementary Material

1 Supplementary Figures and Tables

For more information on Supplementary Material and for details on the different file types accepted, please see { HYPERLINK "https://www.frontiersin.org/guidelines/author-guidelines" \l "supplementary-material" }.

1.1 Supplementary Figures

Supplementary Figure { SEQ Figure * ARABIC }.

Figure legends

(A)

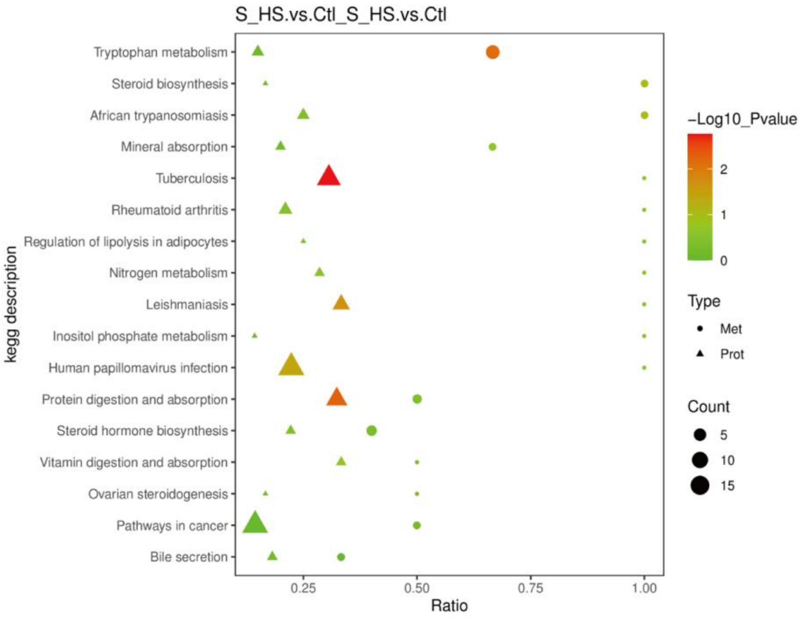

(B)

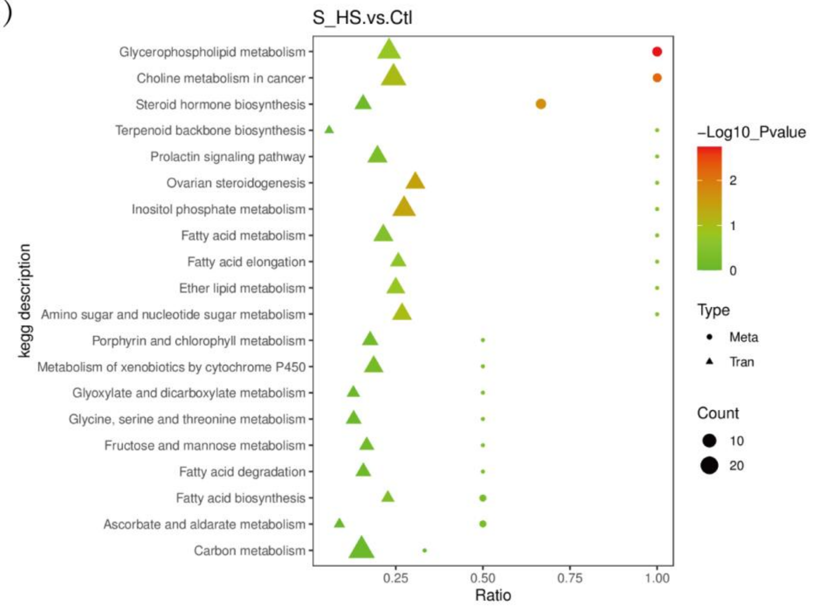

Figure S1. Multi-omics cross-layer analysis of KEGG bubble plots. (A)Metabolomics and Proteomics KEGG Enrichment(S-HS VS CTL);

(B)Metabolomics and transcriptomics KEGG enrichment(S-HS VS CTL)

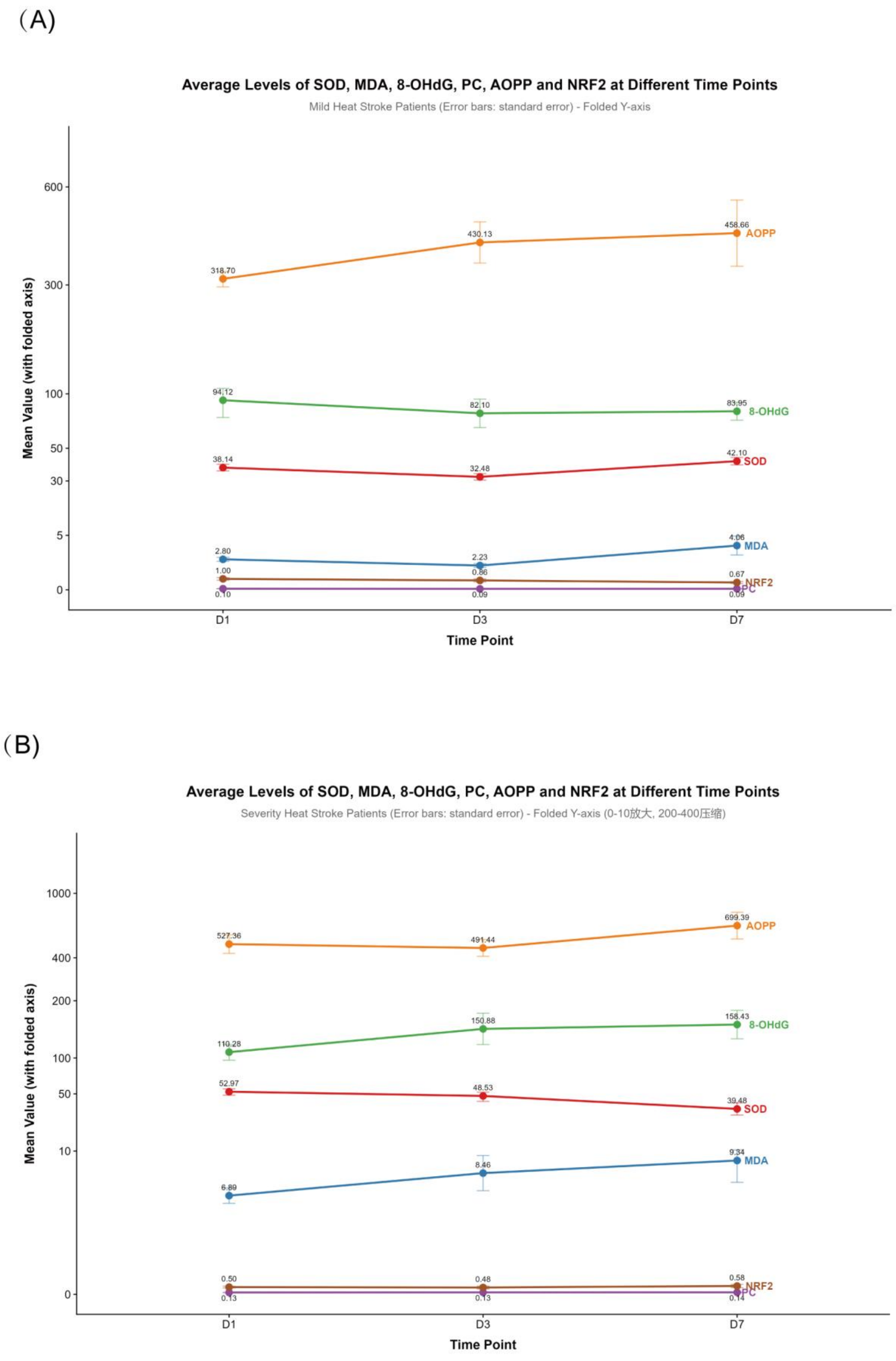

Figure S2. Longitudinal analysis of SOD, MDA, AOPP, PC, 8-OHdG and NRF2 in patients with mild and severe heat stroke

(A)Comparison of SOD, MDA, AOPP, PC, 8-OHdG, and NRF2 levels in patients with mild heatstroke(B)Comparison of SOD, MDA, AOPP, PC, 8-OHdG, and NRF2 levels in patients with mild heatstrokewith severe heatstroke.MDA, malondialdehyde; SOD, superoxide dismutasse; AOPP, Advanced Oxidized Protein Product; PC, Protein Carbonyl; 8-OhdG, 8-Hydroxydeoxyguanosine

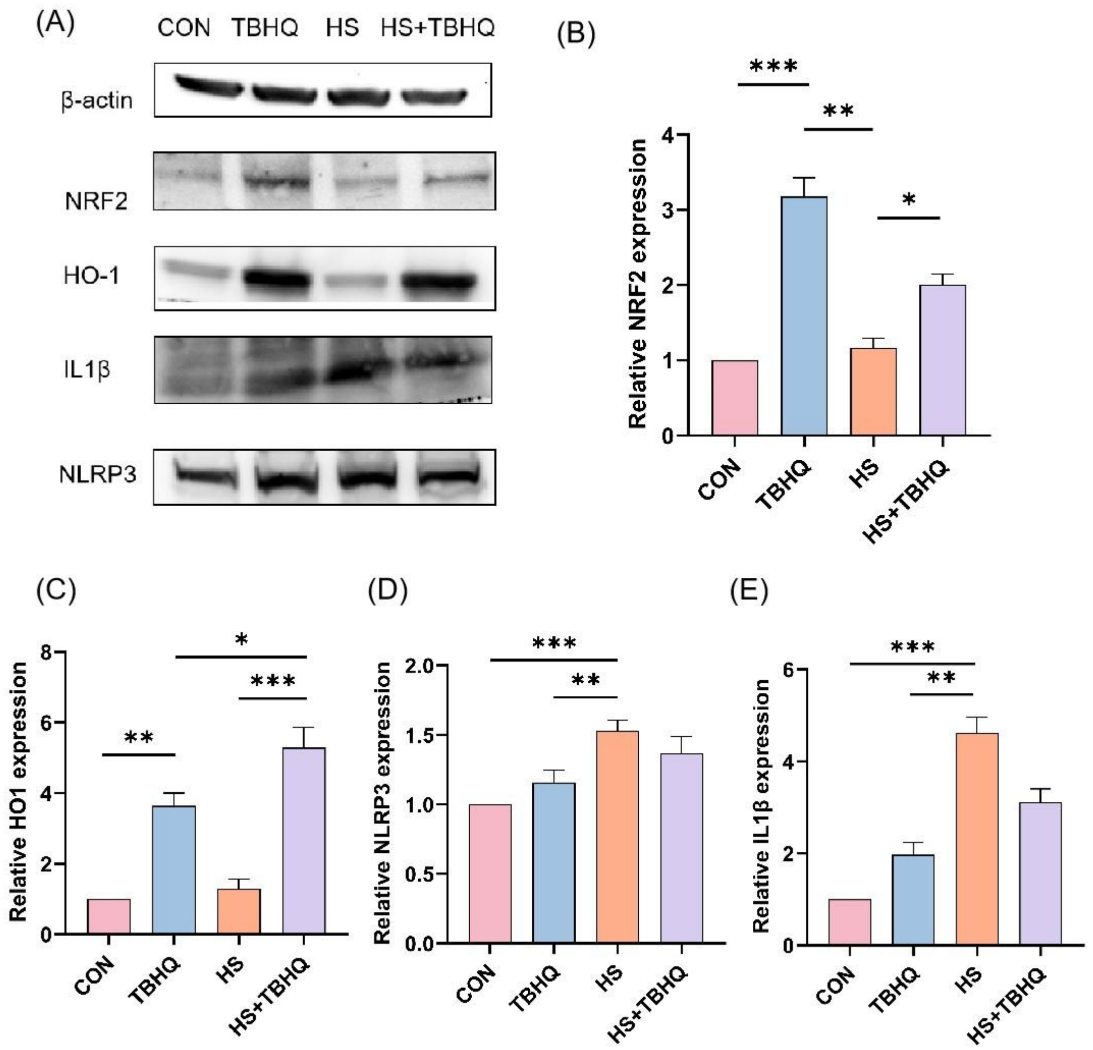

Figure S3. BV2 protein expression level after NRF2 stimulation.(A)Western blotting results .(B) Relative expression level of NRF2 protein；(C) Relative expression level of HO-1 protein；(D) Relative expression level of NLRP3 protein(E)Relative expression level of IL1β protein；NRF2：Nuclear factor erythroid 2-related factor 2;NLRP3：NOD-like receptor family pyrin domain containing 3;HO-1：Heme oxygenase-1;IL-1β：Interleukin-1 beta.*P < 0.05, **P < 0.01, ***P < 0.001.

(A)

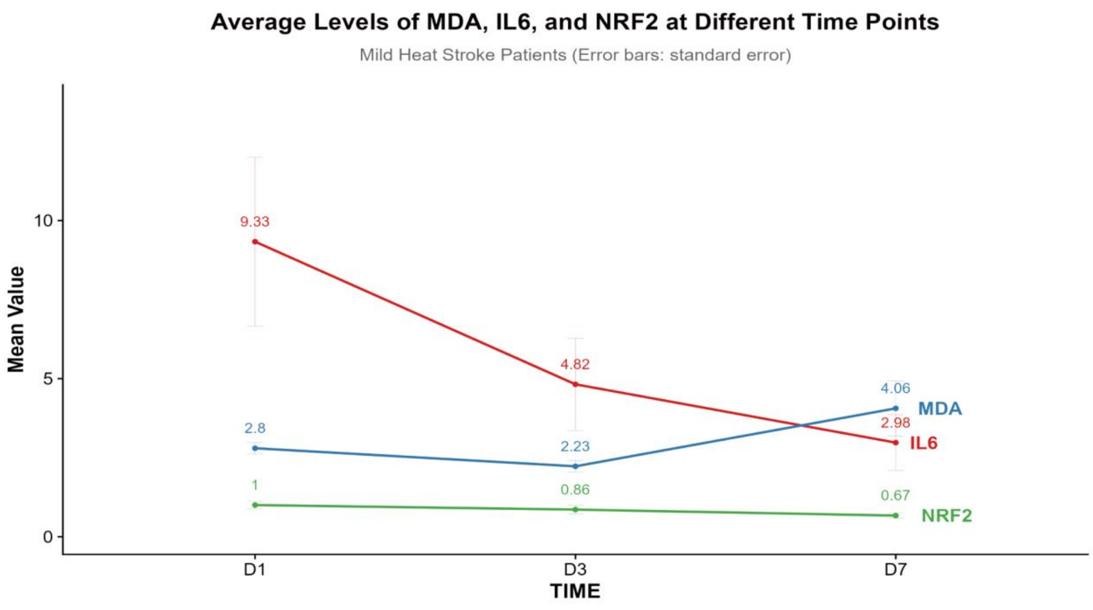

(B)

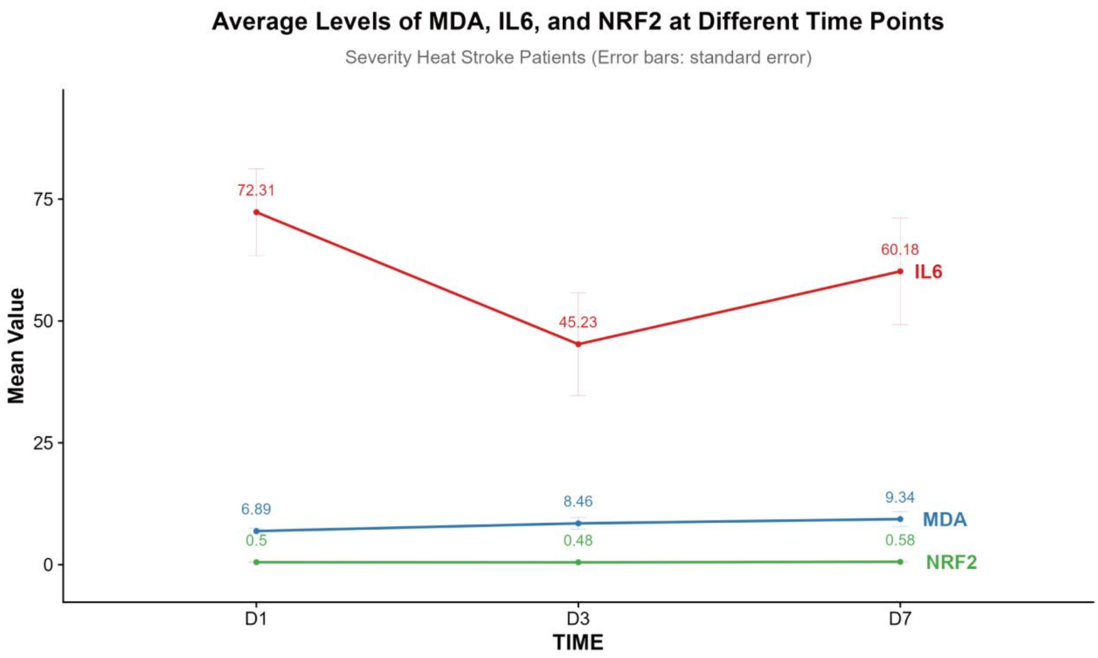

Figure S4. Longitudinal analysis of MDA, IL6 and NRF2 in patients with mild and severe heat stroke(A)Longitudinal analysis of MDA, IL6 and NRF2 in patients with mild heat stroke;(B)Longitudinal analysis of MDA, IL6 and NRF2 in patients with severe heat stroke.MDA, malondialdehyde
